# Successful Local Anesthetic Systemic Toxicity (LAST) Management With Intralipid: A Case Emphasizing Post-stabilization Monitoring and 2020 American Society of Regional Anesthesia and Pain Medicine (ASRA) Updates

**DOI:** 10.7759/cureus.85538

**Published:** 2025-06-07

**Authors:** Amin Farsani, Nicholas K Nuveen

**Affiliations:** 1 Emergency Medicine, Los Robles Regional Medical Center, Thousand Oaks, USA

**Keywords:** asra guidelines, intravenous lipid emulsion (ile), lipid sink mechanism, lipophilic drug toxicity, local anesthetic systemic toxicity (last)

## Abstract

Local anesthetic systemic toxicity (LAST) is a rare but potentially life-threatening condition resulting from elevated plasma concentrations of local anesthetics, with serious consequences including central nervous system (CNS) excitation, cardiovascular collapse, and cardiac arrest. Despite advancements in ultrasound-guided techniques and safer anesthetic protocols, LAST remains a significant clinical emergency, often presenting with symptoms such as seizures, agitation, and tinnitus followed by arrhythmias or bradycardia. Timely recognition and intervention are paramount, with intravenous lipid emulsion (ILE; 20% intralipid) now serving as a cornerstone in the treatment of LAST. ILE functions as a "lipid sink," sequestering lipophilic anesthetics from plasma and restoring hemodynamic stability. This report presents a case of a 67-year-old female who developed LAST after a cervical nerve block with ropivacaine during an outpatient surgical procedure. She experienced a brief tonic-clonic seizure and bradycardia shortly after the block, which resolved following the administration of ILE. Upon arrival at the emergency department, the patient was stable with no recurrent symptoms. The clinical presentation, including the temporal relationship between the anesthesia and the onset of symptoms, supported a diagnosis of LAST, which was further corroborated by normal laboratory values and imaging studies that ruled out other potential causes. The patient was observed for 24 hours and discharged without further complications. This case underscores the importance of early detection, prompt intervention with ILE, and extended post-stabilization monitoring in preventing severe outcomes. It also highlights that while ILE therapy remains highly effective, it requires careful adherence to established dosing and monitoring protocols to manage the risk of biphasic toxicity and other complications. The success of this case reinforces the utility of ILE in treating LAST and supports further research into optimizing its use across various toxicological contexts, particularly in older or high-risk populations. As such, this report advocates for a reevaluation of clinical practices surrounding LAST, with an emphasis on patient-specific considerations and prolonged observation to enhance safety and improve outcomes in the management of this challenging condition.

## Introduction

Local anesthetic systemic toxicity (LAST) is a life-threatening condition resulting from toxic plasma concentrations of local anesthetics. Systemic toxicity from local anesthetics has been estimated to occur in 0.03% of peripheral nerve blocks, or 0.27 episodes per 1000 blocks [1]. In 2022, according to the American Association of Poison Control Centers (AAPCC), 2289 single exposures to lidocaine were reported, along with 2517 single exposures to other or unknown local and/or topical anesthetics [2]. While ultrasound-guided techniques and safer protocols have reduced its incidence, LAST remains a critical emergency characterized by central nervous system (CNS) excitation and cardiovascular depression requiring prompt recognition and intervention [1,3]. Symptoms of the central nervous system, such as agitation, seizures, and tinnitus, often precede cardiovascular instability, including arrhythmias and cardiac arrest [4]. Intravenous lipid emulsion (ILE; 20% Intralipid) should be initiated as per 2020 American Society of Regional Anesthesia and Pain Medicine (ASRA) guidelines, with a bolus of 1.5 mL/kg over one minute, followed by a continuous infusion at 0.25 mL/kg/min for 30 minutes [3].

Management of LAST has significantly evolved, with ILE therapy becoming a cornerstone of treatment. Introduced in the early 2000s, ILE provides a rapid and effective pharmacological antidote by sequestering lipophilic local anesthetics from plasma. This lipid sink mechanism reduces toxic effects and restores hemodynamic stability [5,6]. Additional mechanisms include enhanced myocardial fatty acid metabolism and improved calcium flux in cardiomyocytes. The efficacy of ILE has been demonstrated in clinical and experimental models, including successful resuscitation in bupivacaine-induced cardiac arrest [6]. Recognizing its efficacy, organizations such as ASRA and the Association of Anaesthetists of Great Britain and Ireland (AAGBI) recommend ILE as a first-line therapy for LAST [3,7].

Beyond managing LAST, ILE has shown potential in managing toxicities caused by lipophilic drugs such as calcium channel blockers, beta blockers, tricyclic antidepressants and some organophosphates [8]. This report highlights a case of LAST successfully treated with ILE and discusses its applications, supported by guidelines and evidence from the literature.

## Case presentation

A 67-year-old female with no significant past medical history and no known drug allergies presented to the Emergency Department (ED) complaining of a mild headache after having a seizure, decreased respiratory drive, and bradycardia immediately after receiving a cervical nerve block prior to an arthroscopic surgical repair for a rotator cuff tear at an outpatient surgical center. The patient was premedicated with 2 mg of midazolam and 50 μg of fentanyl prior to the nerve block and then received ropivacaine without epinephrine. The seizure lasted less than one minute, was tonic-clonic in nature, and resolved after a bolus of intravenous lipid emulsion. The patient also received naloxone to reverse the effects of fentanyl and supplemental oxygen to improve the respiratory drive.

**Figure 1:**
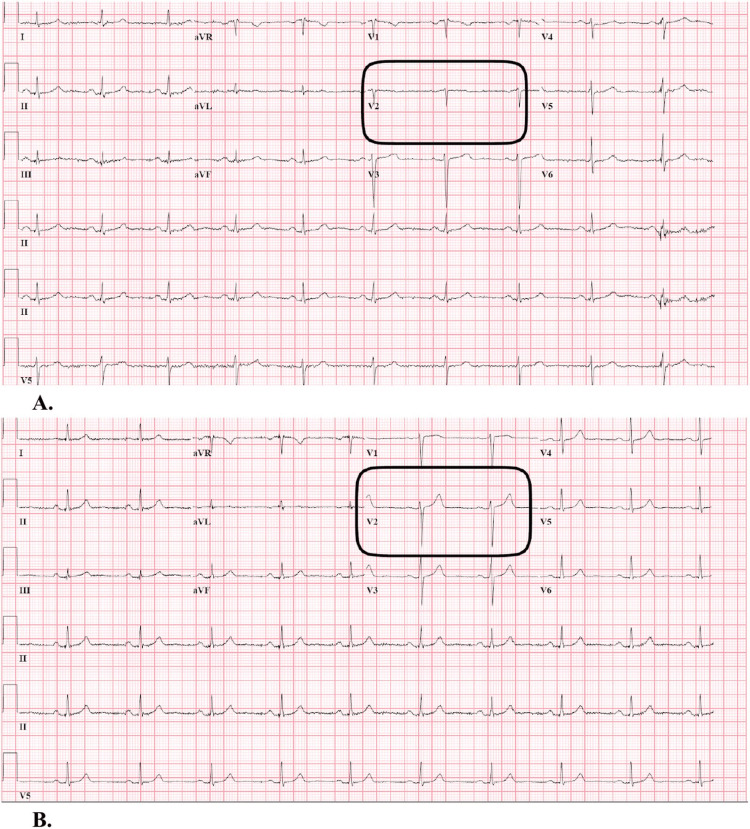
A) Initial ECG performed in the Emergency Department revealing nonspecific T-wave abnormality in lead V2. B) Repeat EKG 17 hours later revealing resolution of nonspecific T-wave abnormality in lead V2.

Upon arrival at the ED, the patient was alert and oriented to person, place, and time but could not recall the event and only remembered waking up with people surrounding her. Vital signs revealed borderline bradycardia at 59 beats per minute, blood pressure 137/81, SpO2 97% on room air, and a respiratory rate of 18 breaths per minute. The physical exam was unremarkable. Differential diagnoses included LAST, anaphylaxis, seizure disorders, or other neurologic events. The complete blood count (CBC), complete metabolic panel (CMP), and lactate were obtained approximately one hour after the seizure-like activity and were within normal limits as depicted in Table 1. The initial electrocardiogram (ECG) revealed a nonspecific T-wave abnormality in lead V2 with resolution on repeat ECG 17 hours later, as depicted in Figure 1. The patient was admitted for continuous cardiac monitoring; no recurrent symptoms were observed and vitals remained stable. Repeat CBC and CMP were unremarkable and CT scan of head without contrast revealed no acute intracranial pathology. The patient was discharged the following day with instructions to follow up with a primary care doctor for further evaluation. 

**Table 1:** Complete blood count, comprehensive metabolic panel and lactic acid results in the emergency department revealing no significant abnormality. BUN: blood urea nitrogen, GFR: glomerular filtration rate, ALT: alanine aminotransferase, AST: aspartate aminotransferase, Hgb: hemoglobin, Hct: hematocrit, MCV: mean corpuscular volume, MCH: mean corpuscular hemoglobin, MCHC: mean corpuscular hemoglobin concentration, RDW: red cell distribution width, Plt: platelet, MPV: mean platelet volume

Lab Test	Lab Value	Reference Range
Sodium	139	136 - 145 mmol/L
Potassium	4.4	3.6 - 5.1 mmol/L
Chloride	107	98 - 107 mmol/L
Carbon Dioxide	26	21 - 32 mmol/L
Anion Gap	6	5 - 15
BUN	10	7 - 18 mg/dL
Creatinine	0.6	0.55 - 1.02 mg/dL
Estimated GFR	98	> 90
Glucose	111	74 - 99 mg/dL
Lactic Acid	0.8	0.4 - 2.0 mmol/L
Calcium	9.5	8.5 - 10.1 mg/dL
Phosphorus	3.6	2.5 - 4.9 mg/dL
Magnesium	2.1	1.6 - 2.6 mg/dL
Total Bilirubin	0.9	0.2 - 1.0 mg/dL
AST	50	10 - 37 IU/L
ALT	30	13 - 56 IU/L
Total Alk Phosphatase	50	45 - 117 IU/L
Serum Total Protein	7.7	6.4 - 8.2 g/dL
Albumin	4	3.4 - 5.0 g/dL
Globulin	3.7	2.5 - 4.0 g/dL
WBC	4.5	4.0 - 11.0 10^3/uL
RBC	3.98	3.8 - 5.2 10^6/uL
Hgb	12.3	11.7 - 15.7 g/dL
Hct	39	37.0 - 47.0 %
MCV	98	82 - 98 fL
MCH	31	27.0 - 34.0 pg
MCHC	31.6	32.0 - 36.0 g/dL
RDW	13.6	11.5 - 14.5 %
Plt Count	290	150 - 400 10^3/uL
MPV	9.5	6.0 - 11.5 fL

In distinguishing LAST from other conditions such as anaphylaxis, seizure disorders, or other neurologic events, several laboratory tests, imaging studies, and clinical features played crucial roles. For example, the patient's CBC, CMP and lactate levels were within normal limits. This helped rule out systemic inflammatory responses or metabolic disturbances often seen in anaphylaxis or sepsis. Anaphylaxis, in particular, is frequently accompanied by hypotension and cutaneous manifestations like urticaria or angioedema, features that were absent in this case. 

Imaging also contributed to the differential diagnosis. A head CT scan without contrast was performed and revealed no acute intracranial pathology, which assisted in excluding structural causes of seizures, such as intracranial hemorrhage or ischemic stroke. In contrast, a seizure disorder might have been supported by abnormal neuroimaging like structural lesions, calcifications, evidence of prior infarcts or hemorrhages, and cortical abnormalities which were not present. 

Clinically, the sequence and timing of symptoms were consistent with LAST. The patient experienced central nervous system symptoms, including agitation and a generalized tonic-clonic seizure, shortly after receiving a local anesthetic injection; however, alternative causes of seizure activity, including direct effects of the anesthetic agent or other metabolic or hemodynamic factors, could not be definitively excluded.

Together, the normal laboratory and imaging findings, combined with the specific timing and pattern of CNS and cardiovascular symptoms, supported the diagnosis of LAST and helped exclude other potential causes such as anaphylaxis, primary seizure disorders, or other neurologic events.

## Discussion

LAST occurs due to the systemic absorption of local anesthetics, which block sodium channels in excitable tissues such as the brain and myocardium. This blockade leads to an initial phase of CNS excitation, often characterized by seizures, followed by cardiovascular depression and potential cardiac arrest [1,3]. Preventive strategies play a crucial role in minimizing the risk of LAST. Clinicians should avoid the rapid injection of large volumes by administering local anesthetics in incremental doses rather than a single rapid injection. This approach allows for the early detection of toxicity symptoms and reduces peak plasma concentrations [3]. The use of test doses is also essential; a test dose of 3 to 5 mL of a short-acting local anesthetic containing epinephrine should be administered before initiating the complete block to detect intravascular or intrathecal injection. If the patient is repositioned, the test dose should be repeated to account for potential catheter displacement [1]. Allowing adequate time between test doses ensures proper monitoring for signs of systemic toxicity or unintended effects before proceeding with additional administration. Additionally, using the lowest effective dose and concentration necessary to achieve the desired clinical outcome minimizes systemic exposure and further reduces the likelihood of toxicity [1]. 

Elderly patients are particularly susceptible to LAST due to reduced hepatic metabolism, altered protein binding, and diminished cardiac and neurological reserve. These factors increase the risk of systemic toxicity, necessitating lower doses and more cautious administration [1,3]. While ropivacaine is considered safer than bupivacaine, it remains capable of causing significant systemic toxicity, particularly when administered in higher doses or without epinephrine. The absence of epinephrine may lead to faster systemic absorption and increased plasma concentrations, elevating the risk of adverse effects [1]. 

Early administration of ILE therapy is critical in the management of LAST. ILE reduces the plasma concentration of lipophilic local anesthetics and restores hemodynamic stability through its lipid sink mechanism. The clinical efficacy of lipid rescue is influenced by pharmacokinetic parameters such as the partition constant and volume of distribution of the toxic agent. These factors determine the ability of the lipid emulsion to sequester and effectively reduce plasma concentrations of the anesthetic, thereby enhancing treatment outcomes [9]. Rapid initiation of ILE, as recommended by the 2020 ASRA guidelines, can prevent progression to severe cardiovascular complications [3,5]. The therapeutic benefits of ILE have been demonstrated in multiple cases, including a report by Rosenblatt et al., who documented the successful use of a 20% lipid emulsion to resuscitate a patient following a presumed bupivacaine-related cardiac arrest, exemplifying its clinical efficacy [10]. 

Extended monitoring following stabilization is vital to detect biphasic toxicity, where delayed symptoms can occur after initial recovery. Current guidelines recommend 12 to 24 hours of observation, particularly after ILE administration, to ensure stability before discharge [3]. In cases refractory to ILE, adjunct therapies such as vasopressors or cardiopulmonary bypass may be considered. Additionally, ILE has shown promise in managing toxicities caused by other lipophilic agents, such as beta-blockers and calcium channel blockers, expanding its utility in clinical toxicology [8].

In our case, the prompt recognition and successful use of ILE therapy in managing LAST are consistent with similar reports in the literature. For example, Rosenblatt et al. described the successful resuscitation of a patient experiencing bupivacaine-induced cardiac arrest with a 20% lipid emulsion, which parallels our case in both the rapid stabilization achieved and the prompt intervention following symptom onset [10]. Similarly, Neal et al. emphasized early intervention with ILE as a critical component in mitigating severe complications from LAST [3]. However, unlike some cases where more pronounced cardiovascular collapse occurred, our patient presented with a milder clinical picture, characterized by a short-lived tonic-clonic seizure and transient bradycardia. These differences may reflect variations in local anesthetic dosage, patient-specific factors, or the speed of intervention. 

Although cardiovascular collapse was not observed in this patient, the administration of ILE was clinically justified by the presence of a tonic-clonic seizure occurring shortly after local anesthetic administration. The ASRA Practice Advisory explicitly recommends considering ILE therapy not only for cardiovascular instability but also for significant central nervous system symptoms consistent with LAST, such as seizures [3,4]. Prompt treatment in this setting is critical because LAST can progress rapidly from central nervous system excitation to life-threatening cardiovascular depression [5]. Recent studies also emphasize that early use of ILE in cases of severe CNS toxicity may prevent further complications and stabilize patients before cardiovascular compromise develops [1]. Therefore, despite the absence of cardiovascular collapse in this patient, the seizure activity and timing after local anesthetic exposure met the criteria for prompt intervention with ILE, aligning with current recommendations and best practices in managing suspected LAST.

Despite the clear temporal relationship and clinical features observed in our patient, it is important to acknowledge the diagnostic uncertainty inherent of seizure-like activity following local anesthetic administration. While the symptoms and timing were consistent with LAST, other possible explanations such as direct sedative effects of the local anesthetic agent itself, metabolic or hemodynamic factors could not be definitively excluded in this setting. The diagnosis of LAST in the emergency department is primarily clinical, based on characteristic symptoms and temporal associations, but confirmatory testing (e.g., plasma local anesthetic levels) is rarely feasible [3]. This reinforces the importance of maintaining a broad differential diagnosis and of early supportive interventions to optimize outcomes, regardless of the underlying cause.

Although the outcome was ultimately favorable, potential pitfalls remain in the management of LAST. One limitation is the possibility of delayed toxicity or biphasic reactions, as highlighted in the literature [1]. In cases where initial stabilization is achieved with ILE, continuous monitoring over a prolonged period is essential, as delayed symptoms may emerge due to redistribution of the anesthetic. Additional concerns include the risk of local tissue injury from repeated injections or the inadvertent administration of excessive lipid, which might lead to metabolic complications. These issues underscore the importance of adhering strictly to established protocols and maintaining vigilance during the post-stabilization period. 

Alternative management strategies have been explored in the context of LAST. While supportive measures such as oxygen supplementation, vasopressor support, and, in extreme cases, cardiopulmonary bypass have been described, these approaches do not address the underlying toxic mechanism as directly as ILE therapy does [6]. The lipid sink effect, which sequesters lipophilic anesthetic molecules and reduces their plasma concentrations, makes ILE therapy the optimal choice in our scenario, as it directly targets the pathophysiological basis of toxicity. Furthermore, systematic reviews have reinforced the efficacy of ILE in various settings of drug toxicity, supporting its role as a first-line treatment in LAST cases [8,11]. 

In summary, while our case shares common features with previous reports of LAST successfully managed with ILE therapy, it also highlights the importance of comprehensive post-stabilization monitoring and the need to be aware of potential complications. Future research should continue to refine dosing protocols and explore adjunctive therapies, ensuring that ILE therapy remains a cornerstone in the management of LAST. 

## Conclusions

This case not only reinforces the efficacy of ILE therapy in managing LAST but also highlights critical aspects of its application that warrant a re-evaluation of current clinical practices. The rapid stabilization achieved with ILE in this case, coupled with the implementation of an extended monitoring protocol, emphasizes the urgent need for early recognition and prompt intervention according to established guidelines. These findings advocate for a closer examination of current practice standards, particularly the duration of post-stabilization monitoring and management strategies for biphasic toxicity, which remains an underexplored aspect of LAST management. Given the unique clinical presentation in this case, which differed from more severe manifestations seen in previous reports, it highlights potential gaps in protocol application, suggesting that the current recommendations for monitoring could be more tailored to specific patient profiles or circumstances.

Furthermore, this case serves as a catalyst for future research aimed at optimizing ILE dosing protocols and refining our understanding of its mechanisms of action in various toxicological scenarios. It opens the door for studies that not only investigate the pharmacokinetic properties of lipid emulsion in LAST but also explore its potential in managing other toxicities involving lipophilic drugs such as calcium channel blockers, beta blockers, and tricyclic antidepressants. By sharing these insights, this report encourages clinicians and researchers to revisit and potentially update guidelines to improve patient safety and clinical outcomes in managing LAST, thereby driving forward the development of more nuanced and evidence-based practices.
